# Microglia in a Dish—Which Techniques Are on the Menu for Functional Studies?

**DOI:** 10.3389/fncel.2022.908315

**Published:** 2022-06-03

**Authors:** Philipp Aktories, Philippe Petry, Katrin Kierdorf

**Affiliations:** ^1^Institute of Neuropathology, Faculty of Medicine, University of Freiburg, Freiburg, Germany; ^2^Faculty of Biology, University of Freiburg, Freiburg, Germany; ^3^Center for Basics in NeuroModulation (NeuroModulBasics), Faculty of Medicine, University of Freiburg, Freiburg, Germany; ^4^CIBSS-Centre for Integrative Biological Signalling Studies, University of Freiburg, Freiburg, Germany

**Keywords:** microglia, *in vitro* models, cell culture systems, primary cell culture, transformed cell lines, organotypic cultures, CNS organoid, ipsc-derived microglia

## Abstract

Microglia build the first line of defense in the central nervous system (CNS) and play central roles during development and homeostasis. Indeed, they serve a plethora of diverse functions in the CNS of which many are not yet fully described and more are still to be discovered. Research of the last decades unraveled an implication of microglia in nearly every neurodegenerative and neuroinflammatory disease, making it even more challenging to elucidate molecular mechanisms behind microglial functions and to modulate aberrant microglial behavior. To understand microglial functions and the underlying signaling machinery, many attempts were made to employ functional *in vitro* studies of microglia. However, the range of available cell culture models is wide and they come with different advantages and disadvantages for functional assays. Here we aim to provide a condensed summary of common microglia *in vitro* systems and discuss their potentials and shortcomings for functional studies *in vitro*.

## Introduction

Research of the last century has unraveled the crucial role of microglia—the resident tissue macrophages of the central nervous system (CNS)—during development, immune defense, and homeostasis, but also in various neuropathologies including Alzheimer’s disease and multiple sclerosis (Prinz et al., 2019; Sierra et al., 2019). The study of neurodegenerative and neuroinflammatory diseases requires reliable *in vitro* systems to model microglia physiology and functionality and to facilitate a successful transfer to animal models and later clinical studies (Timmerman et al., 2018). However, to develop such *in vitro* surrogate systems for microglia has proven to be an extraordinary challenge many research groups around the world have attempted to solve. Some approaches rely on direct primary microglia isolation and purification from different developmental stages, others are based on oncogenically transformed cell lines, or co-cultures such as mixed glial-cultures, which are commonly used (Hansson, 1984; Blasi et al., 1990; Bohlen et al., 2019). Further on, during the last decade induced pluripotent stem cell (iPSC) based models have been developed, including the establishment of 3D CNS organoid cultures (Lancaster et al., 2013; Lancaster and Knoblich, 2014; Ormel et al., 2018), which aim to provide novel, elegant techniques to study brain tissue and also microglia *in vitro*. Although impressive technological advances have led to a deeper understanding of brain resident phagocytes on a single cell level (Hammond et al., 2019; Li et al., 2019; Masuda et al., 2019) there is not yet an ultimate cell culture model available to fulfill all research needs and to overcome shortcomings of maintaining microglia *in vitro*. In this review we want to summarize the available *in vitro* models for microglia which all have certain advantages and disadvantages and highlight which research questions the different models are best suited for.

## Primary Cell Cultures

**Figure 1:**
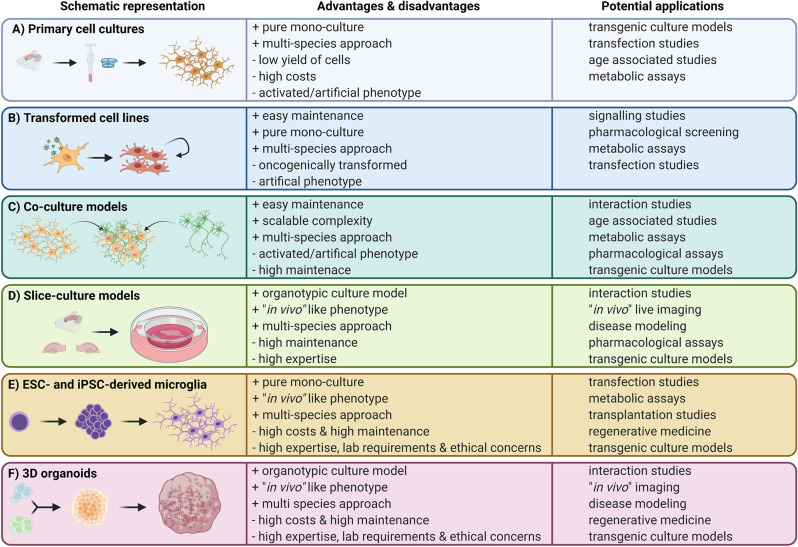
Overview of available microglia *in vitro* approaches. Left panels: schematic representation of the cell culture model; middle panels: advantages and disadvantages of the *in vitro* approach; right panels: potential applications.

**Figure 2:**
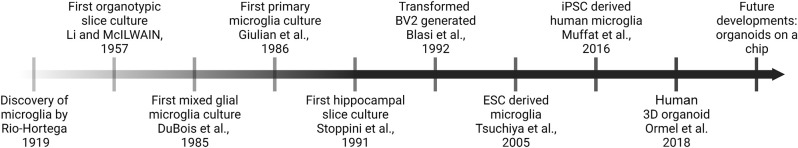
Timeline of selected milestones in the development of microglia *in vitro* models over the last decades.

Cultivation of freshly isolated microglia were thought to represent the closest surrogate system to the *in vivo* conditions (Figure 1). The isolation of microglia from different developmental stages requires the dissociation of the CNS tissue into a single-cell suspension either *via* mechanical dissociation or enzymatic digestion (Haimon et al., 2018; Ocañas et al., 2022), followed by different purification methods such as fluorescence activated cell sorting (FACS; Hickman et al., 2013; Bennett et al., 2016; Pan and Wan, 2020) or magnetic activated cell sorting (MACS; Gordon et al., 2011; Nikodemova and Watters, 2012). These sorting—and dissociation—based cultures offer an easy approach to obtain a pure microglia culture without contamination by other CNS-resident cells. However, sorted cells often develop an altered metabolism and display artificial activation (Gosselin et al., 2017; Haimon et al., 2018; Llufrio et al., 2018; Mattei et al., 2020; Ocañas et al., 2022), increased motility, and altered phagocytosis speeds (Montilla et al., 2020). Even though they cannot model a fully accurate *in vivo* phenotype, they offer a surrogate for many *in vitro* microglia studies. FACS or MACS purification of microglia for cell cultures often results in a low cell number. Pan and Wan reported ~7 × 10^4^ microglia *via* FACS and ~11.7 × 10^4^ microglia *via* MACS sorting (Pan and Wan, 2020). Therefore, primary cultures from MACS or FACS-sorted microglia would require a high number of animals to allow performance of functional assays. Moreover, human brain tissue is very limited, thus functional *in vitro* studies with sorted human primary microglia are barely possible. Culture of sorted microglia from adult CNS tissue does not allow a long-term culture approach, resulting in a short time window for functional assays and screenings. A rather different approach to gain primary microglia is the use of a primary mixed glial culture (DuBois et al., 1985; Murphy et al., 1990; Chen et al., 2013; Figure 2). Here microglia grow on a confluent glial cell layer, and are mechanically shaken off to gain pure microglia in monocultures which can be used for functional assays. Mixed glial cultures are most often generated from neonatal or embryonic CNS tissue, hence the generated microglia have a rather activated and immature phenotype and do not serve as an ideal tool to mimic adult microglia. This further points to the importance for considering the developmental stage of microglia used for the culture system according to the research question. Nevertheless, neonatal microglia from mixed glial cultures are still widely used (Georgieva et al., 2018) offering advantages such as a high cell yield after a short cultivation period. Primary microglia cell culture systems are very liable to various factors including the choice of culture flask coating or the supplements in the media itself such as serum, growth factors, or added metabolites. The media used to culture microglia can be supplemented with various necessary growth factors and cytokines such as macrophage colony stimulating factor (M-CSF) and Interleukin-34 (IL-34; Bohlen et al., 2017), which are important for microglia differentiation (Ginhoux et al., 2010; Greter et al., 2012; Wang et al., 2012) and promote culture growth and survival (Bohlen et al., 2017), or transforming growth factor beta (TGF-β), which has been shown to be an essential cytokine for adult microglia homeostasis and maturation (Butovsky et al., 2014; Zöller et al., 2018). Additionally supplementation with lipids such as cholesterol have also proven to favor microglia survival *in vitro* (Bohlen et al., 2017). Beside media supplementation, various studies were able to demonstrate that different culture coatings such as polyethyleneimine (PEI; Sepulveda-Diaz et al., 2016) and poly-D-lysine (PDL; Lian et al., 2016) have proven to selectively promote microglia attachment and culture growth. PEI coating is a positively charged polymer promoting a high cell yield of pure neonatal microglia cells (Sepulveda-Diaz et al., 2016). In contrast poly-l-lysine (PLL) or PDL coating can be used to not only culture microglia but also neurons and astrocytes (Skaper et al., 2012). On the contrary, these different media conditions also show the impact and artificial modulation of media supplementation on the functionality of the cells such as phagocytic capacity and proliferation (Bohlen et al., 2017), making it difficult to find the right balance between optimal culture conditions and inducing an artificial phenotype. Further shortcomings are the downregulation of important signature markers on primary microglia already hours after culturing them *in vitro* (Bohlen et al., 2017; Gosselin et al., 2017). These markers include microglia signature genes such as *transmembrane protein 119 (Tmem119)* and *purinergic receptor (P2ry12)*. These studies showed that sorted primary microglia undergo detrimental transcriptomic alterations *in vitro*, making it sometimes difficult to use primary microglial cultures for exploring their functions since they do not fully recapitulate the *in vivo* microglia phenotype (Bohlen et al., 2017; Gosselin et al., 2017). For example, Montilla et al. (2020) showed that MACS sorted microglia from rats (P10-P12) lack the ability to degrade and phagocytose myelin *in vitro*, probably due to the reduction of CD68, an important protein associated with phagocytic activity located on the lysosomal membrane. Primary cultures are extensively pushed forward by new approaches to get more organotypic culture conditions. With the available primary microglia monocultures, a lot of various assays are possible such as microglia activation studies and screening for small molecules (Song et al., 2016; Figuera-Losada et al., 2017; Telpoukhovskaia et al., 2020), which all use primary microglia from neonates (P1-P4). Various studies revealed differences between primary cultures and other surrogate systems in distinct functional assays. One study for example showed differences in activation between primary cultured microglia and the murine oncogenically transformed microglial cell line BV2, with a weaker inflammatory response of BV2 cells than primary microglia upon stimulation (Luan et al., 2022). Another advantage of primary microglia is the possibility to culture cells from genetically modified mice and transgenic disease models for neurodegenerative diseases such as Alzheimer’s disease or multiple sclerosis. Moreover, the use of primary cells allows the generation of cell cultures to compare and study microglial functions across various species, including rodents (Bohlen et al., 2017; Montilla et al., 2020), primates (Zuiderwijk-Sick et al., 2007), and humans (Rustenhoven et al., 2016; Tewari et al., 2021), but also primary cultures of zebrafish or leech microglia might be possible in the future. Further on, new and modified primary microglia culture models are still needed to optimize *in vitro* microglia studies and also to facilitate cross-species characterization.

## Oncogenically Transformed Cell Lines

To avoid high costs, low cell numbers and time-consuming isolation and purification protocols of primary microglia cultures, immortalized cell lines were generated, such as the murine BV2 or N9 cells (Figure 1). These cell lines were generated *via* viral transduction of isolated embryonic microglia with different oncogenes such as *v-raf*/*v-myc* (Blasi et al., 1990; Figure 2). Oncogenically transformed cell lines proliferate rapidly with a short generation time and can be sub-cultured indefinitely, thus allowing easy culture maintenance and a high cell yield (Blasi et al., 1990). However, this is often at the expense of an artificial phenotype and an unnaturally high proliferation rate with no contact inhibition (Bocchini et al., 1992; Napoli et al., 2009). Already in the first publications describing the BV2 cell line and its usage for *in vitro* studies, the authors pointed out that these cells rather resemble activated microglia (Bocchini et al., 1992). Of course, transformed microglial cell lines provide certain similarities to their *ex vivo* counterparts such as expression of ionized calcium-binding adapter molecule 1 (Iba-1) and inflammatory cytokine secretion upon lipopolysaccharides (LPS) stimulation (Horvath et al., 2008), as well as reactivity to fibrillar Aβ (1–42; Kopec and Carroll, 1998). Furthermore they are easy to manipulate, and allow big *in vitro* screens due to their unlimited availability (Pluvinage et al., 2019). Unfortunately, the immortalized microglia cell lines also show alterations in biological processes such as loss of contact inhibition, differentially regulated metabolism, and distinct cytokine production dynamics (Horvath et al., 2008; Das et al., 2016; Melief et al., 2016). Additionally, the unnaturally high rate of proliferation always promotes undesired genetic mutations, which could potentially lead to an even more artificial phenotype. Despite all their disadvantages, the BV2 and N9 cell lines are widely used as a first model system to understand molecular mechanisms of microglial immune activation and the underlying signaling pathways, most likely due to the high cell yield and easy maintenance. For example, Duan et al. (2013) revealed an increased synthesis and secretion of pro nerve growth factor (proNGF) by BV2 and N9 cells induced by LPS stimulation *via* Western blot, a method requiring a high number of cells, which no other approach can offer so far. This revealed a potential target for therapeutic manipulation. BV2 and N9 cells were developed almost 30 years ago and there are more than 3,000 publications available up to date using these cell lines. Even though their high proliferation and activated phenotype do not completely resemble adult microglia or aged microglia, they are often used to study microglia functions during neurodegenerative diseases including Parkinson’s disease (Gu et al., 2018). Beside the murine BV2 and N9 cell lines, there are also cell lines for different species available, for example the immortalized (HAPI) cell line derived from neonatal rat brains (Cheepsunthorn et al., 2001) or the transformed human HMO6 cells generated from embryonic telencephalon tissue (Nagai et al., 2001). Nagai et al. (2001) could show that the generated HMO6 cell line showed similar expression profiles upon activation compared to primary human microglia, providing an easy to use model. Moreover, other studies highlight the ability to perform cross species experiments. By transplanting HMO6 cells in a rat middle cerebral artery occlusion model (MCAO), Narantuya and colleagues could demonstrate the importance of transplanted microglia in stroke animals, in showing reduced ischemic deficits and apoptotic cells in rats transplanted with HMO6 (Narantuya et al., 2010). Oncogenically transformed microglial cell lines bridge the gap of a low cell yield for microglial *in vitro* studies, but come with the risk of artificial activation and an altered behavior due to the oncogenic transformation and excessive proliferation rate.

## Co-Culture Models

To address the lack of tissue specific cues and to understand direct cell-cell interactions, co-culture systems have been developed (Figure 1). Co-culture systems are commonly used to provide a more organotypic environment by mimicking direct interactions between cells inside their respective niche, for example microglia with astrocytes, oligodendrocytes, neurons or other CNS associated cells (Roqué and Costa, 2017; Goshi et al., 2020). Co-cultures are often used to model and understand interactions during different neuroinflammatory or neurodegenerative diseases. Due to a vast variety of co-culturing combinations, available protocols, and adjustments, this leads to an open-ended complexity of culture systems used, making it hard to compare and transfer results from one study to the next. Co-cultures offer a strong basis for interaction investigations ranging from synergic molecular interplays during homeostasis to pathological interactions during activation, inflammation, and disease. Without going into the details of the wide spectrum of studies, we would like to point out some studies using complex co-culture systems. As an example, Goshi et al. were able to use a tri-culture model of microglia, neurons and astrocytes to study the neuroinflammatory interplay after LPS stimulation, and identified classical hallmarks in response to LPS (Goshi et al., 2020). Other groups use oncogenically transformed cell lines for co-cultures. For example one study used BV2 cells and PC12 cells as a surrogate for a microglia and neuron co-culture to describe the function of neuron-derived exosomes driving microglia towards a more activated phenotype with an increased release of proinflammatory cytokines (Yin et al., 2020). Further on, more complex co-cultures are available, such as a model to study the interaction of primary murine microglia and T-cells in a biomimetic 3D extracellular matrix (ECM) culture system (Frühauf et al., 2021). In this study, Frühauf et al. (2021) were able to improve survival of T-cells when co-cultured with microglia and provide a basis to study T-cell activation by microglia. Co-culture systems are also often used to study the interaction of CNS-resident cells as well as immune cells in disease models such as brain tumors. Leite and colleagues used a co-culture model comprising human glioblastoma (GBM) cell lines and a human microglia cell line (CHME3) as a model to study their interaction (Leite et al., 2020). They were able to show a higher GBM proliferation and migration rate and further an increased protective effect of microglia to GBM cells pointing to a potential role of microglia promoting their survival and immune escape. Overall co-culture models have been used to study microglial interactions with other cells for years now and with scalable complexity and feasibility they will still be a useful alternative to more complex models in future studies.

## Slice Culture Models

Taking a cell out of its homeostatic tissue niche and placing it into a plastic dish with an artificial environment entails many problems. Thus, the development of organotypic slice cultures (OSCs) from brain tissue in 1957 marked an important milestone for *in vitro* studies of microglia and other CNS cells leaving them in their CNS environment where interactions with neighboring cells can be observed and modulated (Li and McIlwain, 1957; Figure 2). In 1971, Okamoto and Quastel (1973) published a protocol employing cerebellar slices from guinea pigs, which was adapted to a variety of species and proved to be a valuable tool to study myelination, demyelination, and remyelination processes (Jarjour et al., 2012; Doussau et al., 2017). In 1991, Stoppini et al. (1991) developed a protocol for organotypic hippocampal slice cultures (OHSC) that has since then been widely used until this day (Figure 2). This protocol involves culturing hippocampal sections on a semi-permeable membrane creating an interface between air and culture medium. Similar to the concept of OHSCs, organotypic entorhinal slice cultures (OESCs) combine cortex and hippocampus and are ideal to study processes after neuronal axotomies (Del Turco and Deller, 2007). Microglia in OHSCs transition from a reactive and inflammatory to a largely homeostatic phenotype similar to *in vivo* conditions over the course of 3 weeks (Delbridge et al., 2020). OHSC microglia are of mesodermal origin, which together with the preserved cytoarchitecture and the possibility to perform long-term cultures, makes them an interesting *in vitro* alternative to *in vivo* experiments (Figure 1). The drawback of this model is that OSCs require the usage of immature neonatal tissue (P1-P4) as these tissues still have a higher proliferative capacity, which does not represent an adult or aging CNS or even age-associated diseases (Figure 1). Only a few studies described the successful culture of hippocampal slices from adult mice or rats and most of them only for a short period of time (Xiang et al., 2000; Humpel, 2015; Croft et al., 2019). Moreover, OSCs are usually performed with a high serum content (around 25%) which is far from the physiological situation in the brain, hampers reproducibility and renders pharmacologic small molecules tests difficult as these bind to albumin (Croft et al., 2019). Additionally, some reports have shown that this culture system is translatable to human settings allowing the survival of post-mortem or fetal OHSCs for several weeks (Lyman et al., 1991; Eugène et al., 2014). OHSCs harbor the advantage that they can be swiftly prepared from different transgenic lines and are relatively easy to maintain in culture, making them a suitable tool for many labs worldwide to study microglia function and especially their interaction with neighboring CNS cells.

## ESC- and iPSC-Derived Microglia

Embryonic stem cells (ESCs) are pluripotent cells derived from the inner cell mass of the blastocyst, which can be maintained and passaged *in vitro* (Evans and Kaufman, 1981; Figure 1). Since then, numerous studies using ESCs as a tool to differentiate a plethora of cell types *in vitro* opened new avenues and approaches for biomedical studies (Wobus and Boheler, 1999; Cyranoski, 2018; Zakrzewski et al., 2019). Several protocols are nowadays available to differentiate ESCs into neural progenitors, neurons, astrocytes, oligodendrocytes, and also ES cell-derived microglia (ESdM; Tsuchiya et al., 2005; Napoli et al., 2009; Beutner et al., 2010). Tsuchiya et al. (2005) already observed the presence of ESdM next to neural cells derived from embryonic bodies in 2005 (Figure 2). To obtain monocultures of ESdM, ESCs are first expanded before their differentiation into embryoid bodies is induced by withdrawal of leukemia inhibitory factor (LIF). The differentiation of neural progenitors is achieved by changing the ES cell medium to ITSFn medium composed of DMEM/F12 supplemented with insulin, sodium selenite transferrin, and fibronectin. Many studies reported microglia-like cells in the neural or even neuronal differentiated cultures from ES cells as contaminating subpopulations (Tsuchiya et al., 2005; Napoli et al., 2009), other studies showed direct ESdM differentiation protocols (Muffat et al., 2016). All of these studies reported to obtain a high cell density of ESdM coupled with morphology and marker expression profile comparable to microglia *in vivo*, including the expression of macrophage markers such as macrophage-1 antigen (Mac-1) or Iba-1 and a characteristically low expression of the pan-hematopoietic marker CD45. Together with an analogous transcriptomic signature to primary microglia, these similarities seem to make them a great *in vitro* model to study microglia physiology (Figure 1; Beins et al., 2016). The drawback of ESdM is their expensive generation and the limited availability of transgenically modified ESdM (Figure 1). Moreover, similar to BV2 cells and primary neonatal microglia, ESdM also showed differences to their *in vivo* counterparts, for example the production of high levels of inducible nitric oxide synthase (iNOS) in response to interferon gamma (IFN-γ) stimulation which stands in contrast to observations made for microglia in the adult mouse brain (Napoli et al., 2009). Generation of microglia-like cells from ESCs is holding a huge potential for regenerative medicine and thus, the model was soon adapted to human ESCs. However there are ethical concerns regarding the use of human ESCs whereas they need to be directly generated from human embryos (Thomson et al., 1998; Cowan et al., 2004). This constituted a major roadblock for ESCs in clinical approaches making it very challenging to employ human ESC-derived microglia as a possible therapeutic approach and not only as an *in vitro* model for studying microglial function.

To circumvent the ethical concerns and in the attempt of generating patient-specific cell sources, it was a major breakthrough when the first induced pluripotent stem cells (iPSCs) were established. iPSC-derived microglia (IdM) can be obtained by inducing the formation of embryoid bodies from iPSCs and further using a specialized neuro-glial differentiation medium supplemented with M-CSF and IL-34. From this structure, round motile cells expressing microglia surface markers delaminate from the outer border and can be harvested (Muffat et al., 2016; Figure 2). In that way, IdM, at least to some degree, mirror the normal microglia development by passing through a myeloid cell intermediate (Abud et al., 2017). IdM recapitulate both phenotypic and functional characteristics of their *in vivo* counterparts like expression of P2ry12 and Tmem119 (Abud et al., 2017; Quarta et al., 2019). However gene clustering analyses have shown that IdM most closely resemble neonatal microglia rather than adult microglia hence raising the question whether they in fact constitute a model to study adult mature microglia and especially microglia during age-associated neurodegenerative diseases such as Alzheimer’s or Parkinson’s disease (Abud et al., 2017). Even though human IdM allow the unique opportunity to generate large cell numbers from specific patients, iPSC differentiation protocols require a large amount of expertise in cell culture and labor (2–8 weeks depending on species and protocol; Muffat et al., 2016). Nevertheless, iPSCs allow researchers to recapitulate and study microglia with the genetic background of a patient suffering from a specific neurological disorder like Parkinson’s disease and then compare these cells to healthy unaffected donor cells. This unique possibility is not feasible with freshly isolated primary cells from the patients, which are scarcely available and not applicable by using ESdM (Badanjak et al., 2021). Another advantage of the IdM protocols is that the cells can be maintained in serum free culture conditions similar to the endogenous CNS environment and are easier to reproduce, as serum is known to add strong batch effects to a protocol (Figure 1). This model is difficult to employ for the analysis of microglia from transgenic lines as the several weeklong protocol to obtain microglia-like cells from iPSCs needs to be repeated for every transgenic line used (Figure 1). In contrast to primary cells, new iPSCs would need to be transfected for each transgenic line.

## 3D Cerebral Organoids

Recent technical advances in 3D cell culture models have allowed researchers to study the interplay between microglia and other CNS inhabiting cells by using iPSC-derived brain organoids (Figure 1). These surrogate systems can offer the study of molecular interactions of complex neurologic diseases including autism spectrum disorders or microcephaly (Lancaster et al., 2013; Mariani et al., 2015). This revolutionary advancement in creating iPSC-derived brain organoids is reflected by an exponential increase in publications and studies employing this technique over the last 10 years (Pacitti et al., 2019). Cerebral organoids can be derived from either ESCs or iPSCs. Microglia-like cells innately developing in organoid cultures display a typical ramified morphology, which is much more similar to the *in vivo* morphology compared to other cell culture systems. Furthermore, the cells can be monitored to take over many of their usual homeostatic tasks in the brain such as synaptic pruning of neuronal networks or inflammatory cytokine secretion in response to LPS by transcribing high levels of *Il1β* and *Il6* (Ormel et al., 2018; Figure 2). While similar, the described pro-inflammatory cytokine response to LPS challenge is significantly higher compared to adult microglia raising once more the question whether these microglia like cells in brain organoids do indeed resemble mature adult microglia or rather immature developing microglia being in a pre-activated state (Figure 1). Although hierarchical clustering revealed that organoid-derived microglia cluster closer to adult microglia than for example IdM, they start expressing signature markers like P2RY12 and TMEM119 only after more than 100 days making this protocol very long and laborious (Ormel et al., 2018). Nevertheless, organoid cultures represent a fascinating opportunity to study cell-cell interactions of different CNS cells during developmental stages or after genetic modifications (Figure 1). One major challenge of this new technology is to make it reproducible enough with sufficient consistency for standardized drug testing (Tachibana, 2018). Furthermore, not all CNS cells develop easily in cerebral organoids, for example endothelial cells and other cells comprising the neurovascular unit are difficult to introduce in this model (Cakir et al., 2019). The latest technical advancement of 3D cultures are organoids-on-chips which attempt to solve this problem by providing the needed architecture and structural organization of stem cells and differentiated cells on a microfabricated device adapted to dynamically accommodate the respective research question (Karzbrun et al., 2018; Park et al., 2019).

## Conclusion and Outlook

Microglia cell culture approaches have been used for many decades, offering a wide tool box to study molecular mechanisms and cell-cell interactions *in vitro*. Even though researchers can choose from a wide range of models and numerous approaches to maintain rodent as well as human microglia *in vitro*, the available models are still far from offering an ideal surrogate system, perfectly mimicking microglia *in vivo*. Many obstacles, including artificial activation, low cell yield, loss of signature genes and alterations in activation profiles need to be overcome by development of new *in vitro* approaches and improvement of existing culture systems. Furthermore, researchers need to carefully evaluate, depending on their study type, which cell culture system is the most suitable one to answer their questions. Studies exploring age-associated diseases will struggle with cell culture models where microglia display a rather immature phenotype such as those derived from mixed glial cultures, ESCs, iPSCs brain organoids (Figure 1). Technical advances in high throughput approaches and the continuous development of new transgenic animal models to study microglia are supporting the field to employ *in vivo* studies and analysis of freshly isolated microglia. However, there is still a huge demand for microglia cell culture models, especially if it comes to large pharmaceutical screening studies or the understanding of complex signaling pathways. Furthermore, there is constant demand for cell culture models which allow us to efficiently use microglia from scarce specimens such as human tissue samples and moreover an ethical requirement to reduce and refine animal studies. Hence the field is in urgent need to develop new innovative *in vitro* or even *in silico* surrogate systems mimicking the *in vivo* counterparts as closely as possible to avoid artificial results. Improvement of cell culture platforms will further facilitate an easier and more reliable transfer of results from “the dish” into the living model organisms and finally from there into clinical studies. Therefore, it will be the task of future studies and ongoing research to develop and adapt* in vitro* approaches to study microglial function *in vitro* as closely as possible to the *in vivo* situation.

## Author Contributions

PA and PP wrote the manuscript and designed the figures. KK conceptualized, wrote and edited this review. All authors contributed to the article and approved the submitted version.

## Conflict of Interest

The authors declare that the research was conducted in the absence of any commercial or financial relationships that could be construed as a potential conflict of interest.

## Publisher’s Note

All claims expressed in this article are solely those of the authors and do not necessarily represent those of their affiliated organizations, or those of the publisher, the editors and the reviewers. Any product that may be evaluated in this article, or claim that may be made by its manufacturer, is not guaranteed or endorsed by the publisher.
